# Post-Transcriptional Regulation of Viral RNA through Epitranscriptional Modification

**DOI:** 10.3390/cells10051129

**Published:** 2021-05-07

**Authors:** David G. Courtney

**Affiliations:** Wellcome-Wolfson Institute for Experimental Medicine, Queen’s University Belfast, Belfast BT9 7BL, UK; david.courtney@qub.ac.uk; Tel.: +44-2890972713

**Keywords:** virus, RNA, modification, epitranscriptomic, N6-methyladenosine, mapping, 5-methylcytosine, pseudouridine, HIV-1

## Abstract

The field of mRNA modifications has been steadily growing in recent years as technologies have improved and the importance of these residues became clear. However, a subfield has also arisen, specifically focused on how these modifications affect viral RNA, with the possibility that viruses can also be used as a model to best determine the role that these modifications play on cellular mRNAs. First, virologists focused on the most abundant internal mRNA modification, m^6^A, mapping this modification and elucidating its effects on the RNA of a wide range of RNA and DNA viruses. Next, less common RNA modifications including m^5^C, Nm and ac^4^C were investigated and also found to be present on viral RNA. It now appears that viral RNA is littered with a multitude of RNA modifications. In biological systems that are under constant evolutionary pressure to out compete both the host as well as newly arising viral mutants, it poses an interesting question about what evolutionary benefit these modifications provide as it seems evident, at least to this author, that these modifications have been selected for. In this review, I discuss how RNA modifications are identified on viral RNA and the roles that have now been uncovered for these modifications in regard to viral replication. Finally, I propose some interesting avenues of research that may shed further light on the exact role that these modifications play in viral replication.

## 1. Introduction

The post-transcriptional regulation of mRNA function by the covalent modification of individual nucleotides, referred to as epitranscriptomic gene regulation, has attracted increasing interest in recent years. Through the development of better mapping techniques to identify the sites of RNA modification, the ability to quantify these modified residues through mass spectrometry, and the identification of modification writer and reader proteins (Table 1), researchers have been better able to understand the role that these modifications play across the cellular landscape.

RNA modifications include the addition of generally small biochemical groups to adenosine, cytosine, uracil and guanosine, with a methyl group being the most common addition. The most common mRNA modification, m^6^A, comprises an additional methyl group at the N6 position of adenosine and constitutes ~0.4% of all adenosine residues on human cellular mRNA [1]. The second most common modification appears to be a modified uracil residue called pseudouridine, at ~0.3% of all uracil residues on human mRNAs. Recent data have demonstrated that 5-methylcytosine (m^5^C) at ~0.05% is also quite prevalent on cellular mRNAs [1]. 2′O-methylated base modifications (N_m_) are also widespread, where the methyl group is added to the ribose base as opposed to the nucleoside as is the case for the previous three modifications. 2′O-methylation occurs on all four nucleosides on cellular mRNAs. Common 5′cap modification 7-methylguanosine (m^7^G) has also been recently reported to be found internally on cellular mRNAs, though not a great deal is known about the abundance at this early stage [2]. The dimethyl modification N6, N6-dimethyladenosine (N^6,6^A), and N1-methyladenosine (m^1^A) have also been proposed to be present on cellular mRNAs, though if they are indeed present, they are at extremely low levels [3]. Finally, N4-acetylcytidine (ac^4^C) rounds out the list of common mRNA modifications and this arises from the addition of an acetyl group to cytosine. Interestingly, this is the first and, so far, only acetylation event reported on eukaryotic mRNAs [4].

The abundance of these modifications on viral RNA is less well understood. However, in recent years, there have been a growing number of publications investigating the presence of RNA modifications, mostly m^6^A, on RNA from a variety of RNA and DNA viruses, as detailed in Table 2. It appears that m^6^A modifications are highly prevalent on viral RNA, seemingly present on RNA from every virus that has been investigated. Many of the studies compiled in Table 2 identify the presence of RNA modifications by mapping their locations. Three publications, however, performed ultra high-performance liquid chromatography and tandem mass spectrometry (UPLC–MS/MS) to identify pools of RNA modifications in highly purified RNA isolated from HIV-1/MLV virions [5,6], or RNA isolated from positive-strand RNA virus-infected cells [7]. All these studies listed in Table 2 provide us with a clear picture that RNA modifications litter the viral RNA of infected cells.

The presence of these modifications has been found to increase the replication of HIV-1, SV40, HBV and IAV [8,9,10,11,12,13]. However, the exact nature of how RNA modifications confer this advantage remains a mystery. Viral genomes are constantly under an intense selective pressure. Therefore, RNA modifications that confer a selective advantage to viral replication will be selected for and theoretically a virus will quickly evolve to acquire a level of modification that maximizes this advantage. I and others have recently proven this to be the case in two retroviruses, HIV-1 and MLV, where a number of RNA modifications were found on viral RNAs at greater levels than have been observed on cellular mRNAs [5,6,9,10,14]. This conclusion is further supported by a previous study that found an enrichment of a range of RNA modifications on RNA viruses including Zika, Dengue and hepatitis C viruses [4]. The modifications m^6^A, m^5^C, 2′O-methylation and ac^4^C have now been mapped on HIV-1 RNAs [6,9,10,11,14,15], with m^6^A having additionally been mapped on RNA from IAV, MLV, SV40 and a number of flaviviruses [5,6,12,13,16,17].

While the function of these modifications on mRNA is slowly being elucidated, with roles in splicing [18,19,20], translation [21,22,23,24,25,26,27], trafficking [28] and stability [11,29,30,31] all being proposed, whether they have any unique and distinct roles on viral RNA is still as yet unclear. This review will attempt to summarise the current understanding in the field surrounding RNA modifications and their roles in viral replication. I will review the current methods in the field for identifying sites of modification on viral RNA, the stages of the replication cycle seemingly most susceptible to epitranscriptomic mediated alterations, and finally future possible areas of research that could proceed to answer important questions surrounding viral epitranscriptomics.

**Table 2:** A summary of viruses that have previously been reported to carry some of these nine common RNA modifications on virally encoded mRNAs.

Virus	Genome	m^6^A	m^5^C	ψ	m^1^A	ac^4^C	Nm	m^7^G	m^1^G	m^6,6^A
Adenovirus serotype 5	DNA	[18]								
Dengue virus	RNA	[7,16]	[7]	[7]	[7]	[7]	[7]	[7]	[7]	[7]
Enterovirus 71	RNA	[32]								
Epstein–Barr virus	DNA	[33]								
Hepatitis B virus	DNA	[8,34]								
Hepatitis C virus	RNA	[7,16,34]	[7]	[7]	[7]	[7]	[7]	[7]	[7]	[7]
HIV-1	RNA	[6,7,9,10,14,35]	[6,7,36]	[7]	[6,7]	[7,11]	[6,7,15]	[6,7]	[6,7]	[6,7]
Human metapneumovirus	RNA	[37]								
Influenza A virus	RNA	[12,38,39]								
Kaposi’s sarcoma-associated herpesvirus	DNA	[40,41]								
Measles virus	RNA	[42]								
Murine leukaemia virus	RNA	[5]	[5,43]		[5]	[5]	[5]	[5]	[5]	[5]
Poliovirus	RNA	[7]	[7]	[7]	[7]	[7]	[7]	[7]	[7]	[7]
Respiratory syncytial virus	RNA	[44]								
SARS-CoV-2	RNA	[45]	[46]							
Sendai virus	RNA	[42]								
Simian virus 40	DNA	[13]								
Vesicular stomatitis virus	RNA	[42]								
West Nile virus	RNA	[16]								
Yellow fever virus	RNA	[16]								
Zika virus	RNA	[7,16,17]	[7]	[7]	[7]	[7]	[7]	[7]	[7]	[7]

## 2. Viral Modification Mapping

### 2.1. Antibody Mapping

**Figure 1:**
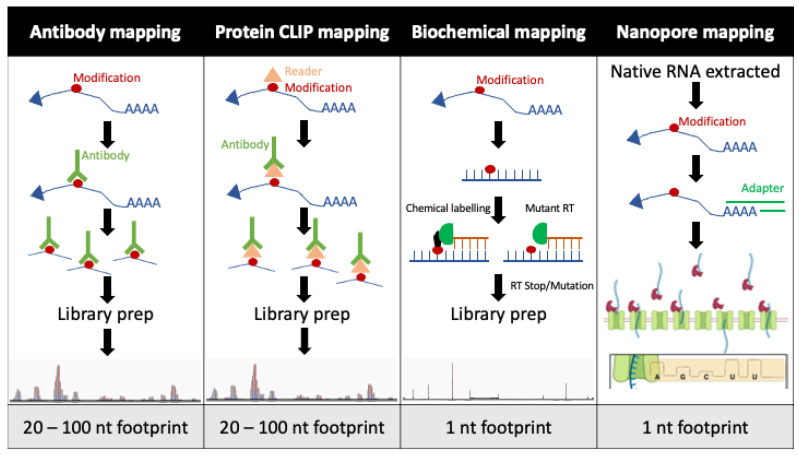
Schematic of the four main methods of mapping RNA modifications. Antibody mapping and protein clip mapping are straightforward techniques involving capture of modified RNA fragments by antibodies before elution and next-generation sequencing, which yields footprints of 20–100 nt. Biochemical mapping generally involves either chemical labelling of a modified residue to block reverse transcription, or a mutant reverse transcriptase that spontaneously stops upon encountering a modified residue. Again, these products undergo next-generation sequencing, but the resultant footprint of these methods is 1 nt. Finally, Nanopore mapping uses a new technique of nucleotide detection by calculating electrical current as the RNA passes through a pore. Each nucleotide alters the electrical current differently, with minor fluctuations also detectable when modified nucleotides are present. This method also results in a 1 nt footprint and is capable of sequencing native RNA.

With the commercial availability of modified nucleoside-specific antibodies, novel mapping techniques started to be developed [47]. This was initially focused on m^6^A identification, before methods were adapted to identify m^5^C, m^1^A and ac^4^C [3,23,48]. In short, these methods generally involve extraction of RNA from the target cells, poly(A) purification of mRNA when it is the target of interest, fragmentation of the RNA, capture of RNA fragments containing a given modification by the modification-specific antibody, capture of the antibody on beads and then isolation of the captured RNAs followed by deep sequencing (Figure 1).

These antibody-based methods, or slightly altered versions of them, have since been used to great effect to map a large number of modifications on RNAs from a whole host of viruses. HIV-1 has been the most extensively studied by this method, with antibody mapping having been used for m^6^A, m^5^C and ac^4^C modification identification [6,9,10,11,14]. Mapping of modifications on MLV, another retrovirus, has been performed for both m^6^A and m^5^C using these methods [5], while influenza A virus (IAV) [12], SV40 [13], Zika virus [17], hepatitis B virus [8] and hepatitis C virus [16] m^6^A modifications have also been mapped in this way.

However, antibody-based mapping of modifications is inherently noisy, with input or IgG controls being required for a number of these methods to remove background signal (Table 3). In addition, this form of mapping results in large footprints of around 20–100 nucleotides, making it practically impossible to determine the exact modified residue on viral or cellular RNA (Table 3). Although antibody-based mapping is relatively quick to perform, unlike some of the other methods, and can at least train a researcher’s eye to a region of interest, it should now be complemented with additional mapping methods to validate any proposed sites of modification.

### 2.2. Protein CLIP Mapping

The use of crosslinked immunoprecipitation can be a useful method for RNA modification mapping if the researcher is aware of modification-specific writer or reader proteins. For example, the YTH domain-containing family of protein including YTHDF1, YTHDF2, YTHDF3, YTHDC1 and YTHDC2 (Table 1) are known to be m^6^A-specific RNA-binding proteins, known as m^6^A ‘readers’ first described by Dominissini et al. [47]. Performing CLIP of these specific proteins can help identify RNA footprints containing an m^6^A residue, to a similar resolution to some antibody-based approaches such as PA-m^6^A-seq (Figure 1) [49]. This CLIP-based approach has been used to good effect for mapping m^6^A modifications on viral RNAs including those of HIV-1 [10,14], IAV [12] and SV40 [13].

In addition to CLIP-seq that uses modification-specific reader proteins, for m^5^C, a highly novel CLIP-based method using the NSUN family of writer proteins has also been described and used for mapping m^5^C on viral RNAs. The m^5^C RNA modification is mediated by the seven members of the NSUN family of methyltransferases, NSUN1 through NSUN7, in addition to the DNA methyltransferase homolog DNMT2 (Table 1) [50,51]. NSUN protein mediated methylation of cytosine uses two highly conserved cysteine residues. One cysteine residue (C321 in NSUN2) forms a transient covalent bond to the pyrimidine base, while the second conserved cysteine residue (C271 in NSUN2) is essential for release of the RNA [52]. Hussain et al. 2013 [53] very cleverly exploited this phenomenon to generate a spontaneously crosslinking NSUN2 mutant (C271A) and then proceeded to overexpress this protein to map NSUN2 targeted cytosine residues by immunoprecipitation and deep sequencing without any need for an actual crosslinking step in the procedure. This also avoids any off-target crosslinking issues as only RNA bound by the NSUN protein, in this case NSUN2, will be covalently bound and appear in the deep sequencing analysis. Colleagues and I followed up on this previous study and exploited the same phenomenon with NSUN proteins to identify NSUN2 as the primary m^5^C writer for HIV-1 RNA and to map sites of m^5^C modification on HIV-1 [3]. This may prove to be a powerful method in the future for both writer identification and m^5^C site validation on both viral and cellular mRNAs.

### 2.3. Biochemical Mapping

Biochemical mapping methods are at present the best option for mapping RNA modifications at single-nucleotide resolution (Figure 1). Although these methods have generally only been utilised for mapping modifications on cellular RNA transcripts, they could theoretically be exploited to map modifications on viral RNAs. This would in turn allow for the quantification of the level of modification occupancy at each residue (Table 3). This is a key attribute of biochemical methods that the above mapping techniques fail to deliver. Below I will describe one commonly utilised biochemical technique for different modifications of increasing interest in the area of viral epitranscriptomics.

One method, termed miCLIP, has been described for the identification of m^6^A sites at single-nucleotide resolution on cellular RNAs [54]. This technique uses an m^6^A-specific antibody UV crosslinked to m^6^A-containing RNA, similar to those described above. This results in the introduction of a single polymorphism by reverse transcriptase, which can be detected and quantified by deep sequencing. This method could easily be translated to the study of viral epitranscriptomics.

RNA bisulfite sequencing is another well-used biochemical method, which has been used to map m^5^C modifications to single-nucleotide resolution on cellular RNAs. By this method, RNA is denatured and incubated at a high temperature with sodium bisulfite to chemically deaminate all unmethylated cytosine residues to uracil [55]. This is due to the low reactivity of m^5^C with HSO_3_. Cytosine residues that are ‘protected’ from deamination can then be detected by standard sequencing techniques. However, one drawback of this technique is that protection from deamination can be due to the presence of not only m^5^C residues but further oxidised forms of cytidine including 5-hydroxymethylcytidine (hm^5^C) and 5-formycytidine (f^5^C). Unfortunately, the efficiency of bisulfite conversion is affected by RNA secondary structure. This may be a problem for the use of this technique to map modifications on viral RNAs, which are notoriously rich in secondary structures and may produce too many artifacts [56]. This will have to be tested experimental before one can know for certain.

The ψ-seq technique has been well described for the mapping of ψ residues on eukaryotic cellular RNA [57,58]. This protocol uses *N*-cyclohexyl-*N′*-(2-morpholinoethyl) carbodiimide metho-*p*-toluenesulfonate (CMC) to selectively modify ψ residues. This large CMC modification on each ψ results in a total block to reverse transcription and these prematurely stopped cDNA fragments can be deep sequenced and identified bioinformatically (Figure 1). An identical non-CMC control sample is processed in parallel to determine background levels of premature stopping. This technique again provides the researcher with single-nucleotide resolution mapping of ψ residues; however, this method is still to be tested for mapping ψ modifications on viral RNAs.

Two methods to accurately map the location of Nm base modifications are RiboMethSeq [59] and Nm-seq [60]. RiboMethSeq is a straightforward method of using alkaline fragmentation on an RNA pool, where Nm residues are generally resistant to fragmentation. In short, this fragmented pool is then ligated to adapters and processed for Illumina sequencing following standard protocols. If the sequencing is performed to a great enough depth, underrepresented sites of fragmentation can be identified bioinformatically and it can be surmised that they have arisen due to the presence of Nm residues [59]. By this method, single-nucleotide resolution can be achieved as well as modification occupancy frequency (Table 3). Nm-seq is a more time-consuming approach, though requiring much less read depth. Nm-seq relies on performing multiple oxidation–elimination–dephosphorylation cycles, where, every cycle, an unmodified nucleotide is eliminated from an RNA string unless it is Nm modified and thus protected from elimination. This approach is then coupled with Illumina sequencing where an adapter is ligated to the immediate 3′ residue of the RNA string, which has been enriched for Nm modified nucleotides [60]. In this way Nm residues can be identified by deep sequencing followed by bioinformatic detection of overrepresented residues at the 3′ end of reads, indicating protection from oxidation and thus likely the presence of an Nm residue. At present, only RiboMethSeq has been shown to be effective in detecting Nm residues in viruses, with this approach having been used to great effect on HIV-1, as will be described later [15]. However, I do not foresee any issues with exploiting either sequencing method for the detection of Nm modifications on viral RNAs in future studies.

In addition to those described above, a number of additional biochemical methods have recently been published for mapping modifications including ac4C [23,61,62], m^7^G [2] and m^1^A [63], all of which it is possible to imagine can be translated to the study of viral epitranscriptomics.

### 2.4. Nanopore Mapping

The advent of direct RNA sequencing through Nanopore technology is a particularly exciting advance in the field of epitranscriptomics, and particularly in regard to viral RNA as one can now sequence native viral RNA harvested from isolated cellular compartments as well as purified virions (Figure 1; Table 3). This allows researchers the opportunity to determine whether the modification landscape of viral RNA is consistent throughout the cell, or whether these sites of modification are dynamic. For instance, are modified residues modified throughout the entire viral replication cycle, such as with an influenza vRNA transcribed in the nucleus, trafficked through the cytoplasm, packaged at the cellular membrane, encapsulated in a virion, and upon infection trafficked again through the cytoplasm to the nucleus? Or perhaps only a subpopulation of viral RNA is modified, and this aids in distinguishing viral RNA to be trafficked and packaged versus translated, as could be the case for some positive-strand RNA viruses.

In fact, nanopore-based direct RNA sequencing was recently used for modification identification early in the COVID-19 pandemic by Kim et al. 2020, where the authors identified potentially at least 41 sites of modification on SARS-CoV-2 viral RNAs [46]. One particularly interesting observation in this study regarding viral transcripts was the discovery that modified viral RNA had shorter poly(A) tails than their unmodified counterparts, with the authors proceeding to speculate that the presence of internal modifications could affect viral RNA stability, but further work will be required to elucidate such a proposed mechanism [46].

## 3. Viral RNA Trafficking

The correct trafficking of viral RNA to sites of replication, translation and packaging is critical to the successful completion of the viral replication cycle. RNA modifications have been identified in a number of cases as having an important role in this process of RNA trafficking. Gokhale et al. [16] investigated the role of m^6^A modification on hepatitis C virus (HCV), while also mapping sites of modification on a range of other flaviviruses. These authors found that m^6^A modification had a direct effect on viral RNA retention in virus replication factories, effectively slowing down the infection and potentially leading to a prolonged chronic infection as is characteristic of HCV infection in the liver. However, when m^6^A was depleted, these viral transcripts are more readily bound by viral Core protein and are successfully trafficked to sites of virion packaging within the cell [16]. Two further studies investigated similar dynamics of viral RNA trafficking, but with retroviruses, and found that, in this instance, RNA modifications contributed a positive effect to viral RNA trafficking. Lichinchi et al. [9] explored the effect of m^6^A on HIV-1 and found that the presence of two m^6^A sites in the Rev-response element (RRE) increased the affinity of Rev for the RRE that in turn increased the nuclear export of RRE containing HIV-1 RNA. While Eckwahl et al. [43] focused on m^5^C modification of MLV RNA, which the authors found, through an association with ALYREF, also increased nuclear export of viral RNA and thus increased viral replication. The differing roles for RNA modifications in the trafficking of HCV and retrovirus RNA further adds a fascinating layer to the complexity of modification mediated post-transcriptional regulation of viral RNA.

## 4. Degradation of Viral RNA

RNA modifications, most notably m^6^A, have been shown to dysregulate the stability of cellular mRNAs generally through interaction with the YTH domain-containing family of proteins [30]. This has been shown to be the case with HBV and KSHV RNA, where both sets of authors demonstrated through global depletion of m^6^A by methyltransferase knockdown or simply depletion of YTHDF proteins by siRNAs, that m^6^A contributed to the destabilisation of viral RNA mostly likely through interactions with YTHDF proteins [8,41]. However, in the case of HIV-1 and IAV, this m^6^A induced RNA destabilization does not appear to be the case. The presence of m^6^A residues on the RNA of HIV-1 [10] and IAV [12] have been shown to increase RNA stability and in both these studies authors suspect this is due to an interaction with YTHDF proteins, primarily YTHDF2. This stabilization was most apparent in YTHDF2 tethering experiments in a mammalian expression system by Kennedy et al. where YTHDF1, 2 and 3 proteins where tethered to a luciferase reporter mRNA by MS2 hairpins in the 3′UTR [10]. This interaction was found to increase the luciferase activity by approximately 3–4 fold for each YTHDF protein.

Aside from m^6^A, a recent study by Tsai et al. investigating the role of ac^4^C modifications in HIV-1 RNA found that these modifications to increase the stability of HIV-1 RNA [11]. This finding is supported by previous work surrounding ac^4^C that found a similar phenotype on cellular mRNAs [23]. The authors demonstrate this finding through acetyltransferase knockout and mutagenesis of modified sites on viral RNA. At present, no RNA-binding proteins specific to ac^4^C are known so the authors were unable to speculate as to whether this increase in RNA stability is due to RNA structural changes or RNA-protein interactions.

## 5. Splicing of Viral RNA

RNA modifications have been implicated in the alternation of splicing events for both HIV-1 and adenovirus RNA [6,18]. Regarding HIV-1, colleagues and I reported that m^5^C is generally present at specific locations across the HIV-1 mRNA genome [6]. However, when m^5^C modification was perturbed due to writer knockout or mutagenesis to prevent modification, alternative splicing at one specific site, namely the D1/A2 splice junction, was altered. Interestingly, this reduction in splice acceptor usage was found for both early (~1.8 kb) and late (~4 kb) HIV-1 classes of transcripts. For adenoviral RNA, Price et al. investigated the role of m^6^A modifications on viral splicing [18]. These authors found that depletion of m^6^A modifications globally, by siRNA mediated knockdown of METTL3 expression, significantly reduced the expression of specifically late adenoviral transcripts. They went on to determine that this phenotype was caused by a reduction in splicing efficiency. These studies imply that the presence of RNA modifications, which are already known to affect splicing on cellular mRNA [64], are being utilised to also alter viral RNA splicing patterns.

## 6. Immune Evasion by Viral RNA

This idea of RNA modifications preventing innate immune sensors from recognising foreign RNAs is not a new concept [65,66]. Karikó et al., and more recently Durbin et al., published studies detailing mechanisms by which innate immune sensors may be blocked from recognising foreign RNA if nucleosides within the RNA are modified. Karikó et al. focused on Toll-like receptors (TLRs), while Durbin et al. investigated the immuno-activating conformational change of RIG-I. Both studies found that the presence of m^6^A or ψ diminished the innate immune signalling by TLRs and RIG-I, respectively. However, it should be noted that both these studies used RNAs with high levels of modified nucleosides much greater than would be physiologically relevant for viral RNAs.

However, in what feels like a seamless follow on to this thought-provoking work, one exciting study into the role of RNA modifications in the viral replication cycle was recently published by Ringeard et al. [15] looking into 2′-O-methyl modifications on HIV-1 RNA. This research provided clear evidence that 2′-O-methylation of HIV-1 gRNA by the methyltransferase FTSJ3 prevents recognition of gRNA by the innate immune sensor MDA5 [15]. Through preventing the addition of 2′-O-methyl marks to HIV-1 gRNA by siRNA mediated FTSJ3 knockdown, the authors show that incoming gRNA induces IFN-α and IFN-β expression. Since this work was published four further studies exploring innate immune sensing of viral RNA were published by Lu et al. [37], Chen et al. [35], Kim et al. [34] and Lu et al. [42] demonstrating, in a similar manner to Ringeard et al., that HMPV, HIV-1, HBV/HCV, VSV, MeV and SeV RNAs are also modified to avoid detection by the host cell. In each of these cases, the viral RNA of each virus is m^6^A modified and this is found to prevent recognition by RIG-I, in validation of the phenomenon described previously by Durbin et al. These studies perform experiments to reduce the modification of virally encoded adenosines using methods such as mutation of the viral genome or treatment with 3-deazaadenosine (DAA), an inhibitor of S-Adenosylhomocysteine (SAH) hydrolase and find that this in turn increases the cellular type 1 interferon response to infection.

## 7. Future Avenues of Research

As more research is performed and published surrounding viral epitranscriptomics there are several ways in which scientists in the field can improve on our current knowledge base. First, we need to expand our interests beyond m^6^A and into other common modifications already found on cellular mRNAs. Lead candidates would include m^5^C, ψ and Nm residues, of which very little is currently known about in regard to viral RNA [5,6,15,36]. When investigating how prevalent these modifications are and where they are on viral RNA, we should seriously consider applying multiple mapping methods to increase our confidence in every site of modification being identified. Antibody-based mapping is being used readily as it is a fast and relatively straightforward approach, but it is also error prone due to off target binding leading to faulty mapping data. As stated above, antibody-based mapping also can only provide a footprint of where a modification may be located of approximately 20–100 nucleotides. However, if coupled with biochemical methods, such as those described above, we can map modifications on viral RNA to single base resolution and at the same time be inherently more confident that these are indeed sites of modification. With this data in hand, we can more reliably design hypo-modified viruses through silent mutagenesis to better grasp the phenotypes that arise due to the presence of individual modifications on a viral RNA.

Another methodology for the study of viral epitranscriptomics that may be better utilised in the future is the generation of viral stocks where the genomic RNA is entirely unmodified. This would help answer important questions about the role of modifications during the initial stages of infection, prior to transcription. This concept has rarely been utilised so far [15], but this author believes it could be an extremely useful technique for all RNA viruses, especially as the role of RNA modifications in immune evasion is better investigated.

As the research progresses, we also need to do better at identifying the exact writer protein for the viral RNA being investigated (Table 1). Regarding m^6^A, the writer complex for viral RNA is almost always METTL3/METTL14/WTAP, but for the likes of m^5^C, it is not as straightforward as the writer may be one out of a family of NSUN proteins or even DNMT2. Through the identification of the correct writer protein, the sites of modification can be better validated, by knocking out or knocking down expression of the writer and then remapping the modification on viral RNA. If the correct writer is no longer present, it would be expected that modification would be ablated. The identification of the primary writer protein will also allow researchers to better investigate the relationship between these proteins and the viral polymerases. A number of RNA modifications have been found to be added co-transcriptionally to cellular mRNAs. Therefore, it is a valid hypothesis that the polymerases of viruses that exploit RNA modifications would associate with writer machinery. This is an area of study that is significantly lacking but may reveal surprising discoveries in the coming years that could impact on our knowledge of both viral and cellular RNA modification utilisation. If these potential interactions are proven to be essential to viral replication, this would also be an interesting avenue of research into the development of antivirals.

The field of viral epitranscriptomics is just at the cusp of a research explosion over the next few years as the line that separates molecular virologists and RNA biologists begins to blur. Exciting questions, as discussed above, will be answered in the coming years and we will hopefully establish the key roles for individual RNA modifications in the replication cycles of a wide variety of viruses. Technological advances will only aid in our understanding of these modifications, with enhanced mapping techniques and better quantification of virus modified sites hopefully not far off.

## Figures and Tables

**Table 1:** A summary of writer and reader proteins for common mRNA modifications and the proposed roles for these modifications on human cellular mRNAs.

Modification	Writers	Readers	*Roles on mRNAs*
N6-methyladenosine (m^6^A)	METTL3 METTL4 METTL16	YTHDF1-3 YTHDC1-2	Splicing Stability Translation Localisation
5-methylcytidine (m^5^C)	NSUN2 DNMT2	YBX1	Splicing Translation
2ʹO-methylated nucleosides (Nm)	FTSJ3	Unknown	Structure Stability
N^4^-acetylcytidine (ac^4^C)	NAT10	Unknown	Stability Translation
Pseudouridine (ψ)	PUS7 TRUB1 DKC1	Unknown	Codon misreading
7-methylguanosine (m^7^G)	METTL1	Unknown	Translation
N^1^-methyladenosine (m^1^A)	TRMT6/61A	YTHDF1-3	Translation
1-methylguanosine (m^1^G)	TRMT10A/B	Unknown	Unknown
N6,N6-dimethyladenosine (m^6,6^A)	Unknown	Unknown	Unknown

**Table 3:** A summary of the advantages and disadvantages of the forms of mapping techniques described in this review.

Mapping Method	Advantages	Disadvantages
Antibody mapping	Fast, straightforward technique Can be used to map modifications on lowly expressed RNA	Large footprint of ~20–100 nucleotides Can generate mapping artifacts
Protein CLIP mapping	Quite straightforward Modification specific	Large footprint of ~20–100 nucleotides Must know the writer or reader protein of interest prior to mapping Can generate mapping artifacts
Biochemical mapping	Single-nucleotide resolution Can be used to quantify modification occupancy at specific residues	Can require very large read-depth May not pick up lowly expressed RNAs lower, more technically difficult technique Can generate mapping artifacts
Nanopore mapping	Can map modifications on native RNA	Difficult to differentiate between modifications at present Can generate mapping artifacts

## Data Availability

Data sharing not applicable.
